# Acyl modifications in bovine, porcine, and equine ghrelins

**DOI:** 10.3389/fendo.2024.1411483

**Published:** 2024-05-17

**Authors:** Takanori Ida, Hatsumi Tominaga, Eri Iwamoto, Akito Kurogi, Ayaka Okura, Kengo Shimada, Johji Kato, Atsutoshi Kuwano, Hirotaka Ode, Sayaka Nagata, Kazuo Kitamura, Takashi Yazawa, Miho Sato-Hashimoto, Masahiro Yasuda, Mikiya Miyazato, Yuki Shiimura, Takahiro Sato, Masayasu Kojima

**Affiliations:** ^1^ Division for Identification and Analysis of Bioactive Peptides, Department of Bioactive Peptides, Frontier Science Research Center, University of Miyazaki, Miyazaki, Japan; ^2^ Miyazaki Prefecture Industrial Technology Center, Miyazaki, Japan; ^3^ Clinical Research Center, Kurume University Hospital, Fukuoka, Japan; ^4^ Equine Research Institute, Japan Racing Association, Tochigi, Japan; ^5^ Racehorse Clinic, Ritto Training Center, Japan Racing Association, Shiga, Japan; ^6^ Department of Food Science and Technology, Faculty of Health and Nutrition, Minami Kyushu University, Miyazaki, Japan; ^7^ Department of Projects Research, Frontier Science Research Center, University of Miyazaki, Miyazaki, Japan; ^8^ Department of Biochemistry, Asahikawa Medical University, Hokkaido, Japan; ^9^ Department of Animal Pharmaceutical Science, School of Pharmaceutical Sciences, Kyusyu University of Medical Science, Miyazaki, Japan; ^10^ Laboratory of Veterinary Anatomy, Faculty of Agriculture, University of Miyazaki, Miyazaki, Japan; ^11^ Molecular Genetics, Institute of Life Sciences, Kurume University, Fukuoka, Japan

**Keywords:** ghrelin, acyl-modification, GHS-R, n-octanoic acid, n-butanoic acid

## Abstract

Ghrelin is a peptide hormone with various important physiological functions. The unique feature of ghrelin is its serine 3 acyl-modification, which is essential for ghrelin activity. The major form of ghrelin is modified with n-octanoic acid (C8:0) by ghrelin O-acyltransferase. Various acyl modifications have been reported in different species. However, the underlying mechanism by which ghrelin is modified with various fatty acids remains to be elucidated. Herein, we report the purification of bovine, porcine, and equine ghrelins. The major active form of bovine ghrelin was a 27-amino acid peptide with an n-octanoyl (C8:0) modification at Ser3. The major active form of porcine and equine ghrelin was a 28-amino acid peptide. However, porcine ghrelin was modified with n-octanol (C8:0), whereas equine ghrelin was modified with n-butanol (C4:0) at Ser3. This study indicates the existence of structural divergence in ghrelin and suggests that it is necessary to measure the minor and major forms of ghrelin to fully understand its physiology.

## 1 Introduction

Ghrelin is a peptide hormone purified from the stomach as an endogenous ligand of the growth hormone (GH) secretagogue receptor (GHS-R) (1). A unique feature of ghrelin structure is the modification of its Ser^3^ residue with n-octanoic acid. Ghrelin has been identified in various mammals, birds, reptiles, amphibians, and fish, all of which have a third serine or threonine residue modified with n-octanoic acid (2–10). This octanoyl modification is essential for receptor binding and the subsequent expression of biological activity. The ghrelin receptor GHS-R is highly conserved from fish to humans and is widely expressed in central and peripheral organs, such as the brain, pituitary gland, and pancreas (11–16). Therefore, the ghrelin-GHS-R system is involved in the regulation of various physiological functions, such as GH secretion (1), feeding (17), body temperature (18), gastric motility (19), gastric acid secretion (20), insulin and gastrin secretion (21), circulatory systems (22, 23), and stress responses (24). Recently, an enzyme catalyzing the acyl modification of ghrelin was discovered and named ghrelin-O-acyltransferase (GOAT) (25). As the dynamics of GOAT determine ghrelin activity, its discovery has significantly improved our understanding of the biosynthesis and secretion mechanisms of ghrelin. The secretory regulation of ghrelin by GOAT is the only such mechanism in mammals. Although GOAT can transfer fatty acids ranging in size from acetic to palmitic acid onto des-acyl ghrelin, it prefers medium-chain fatty acids as substrates (26). The main acyl-modified and most potent active form of ghrelin is n-octanoyl ghrelin; however, other medium-chain fatty acids have also been detected as endogenous ghrelin forms. For example, n-decanoyl ghrelin has been purified from the stomachs of frog and bird at levels comparable to those of n-octanoyl ghrelin (27, 28). Moreover, calcium mobility assays using ghrelin synthesized with fatty acids of various lengths have revealed that ghrelin is less active when modified with short-chain fatty acids (29).

Various structural variations have been observed not only in rat and human ghrelins (30, 31) but also in non-mammalian vertebrates, such as rainbow trout, chicken, and bullfrog ghrelins (4, 6, 28). However, the underlying mechanism by which ghrelin is modified with various fatty acids remains unclear. It is important to clarify growth mechanisms in animals from the context of efficient food production and animal models. However, little is known about the endocrine effects of ghrelin on domestic animals. Owing to the diversity of ghrelin structure, particularly its fatty acid modifications, the blood levels of ghrelin may not be measured accurately. In the present study, we purified ghrelin from bovine, porcine, and equine stomachs and identified various types of ghrelin molecules.

## 2 Materials and methods

### 2.1 Construction of an assay system using GHS-R1a-expressing cells

Full-length cDNA of the mouse GH secretagogue receptor (GHS-R1a) (GenBank accession number NM_177330.4; residues 201–1377) was obtained via RT-PCR using mouse hypothalamus cDNA as the template. The sense and antisense primers were 5′-caccctcctcaggggaccagattt-3′ and 5′-aatgagcgatgacggagagat-3,’ respectively. The amplified cDNA was ligated into the pcDNA3.2/V5/GW/D-TOPO Expression Kit (Invitrogen, Tokyo, Japan). The expression vector, mouse GHS-R-pcDNA3.2, was transfected into Chinese hamster ovary (CHO) cells. Stably expressing cells were selected using 1 mg/ml G418 (Nacalai Tesque, Kyoto, Japan). The selected cell line, CHO-mouse GHS-R1a-line 24–5, showed the highest expression of mouse GHS-R1a mRNA. Cells were cultured in a humidified environment with 95% air and 5% CO_2_. Changes in intracellular Ca^2+^ concentrations ([Ca^2+^]_i_) were measured using a FlexStation 3 fluorometric imaging plate reader (Molecular Devices, CA, USA). CHO-mouse GHS-R1a cells (3 × 10^4^ cells) were plated in 96-well black-walled microplates (Corning, NY, USA) for 20 h before each assay. The cells were incubated with 100 µL of Calcium 4 assay kit (Molecular Devices) for 1 h, and then 50 µL of each sample was added to the CHO-mouse GHS-R1a cells. The maximum [Ca^2+^]_i_ change was determined as the response.

### 2.2 Purification of ghrelins

Bovine (Japanese black breed, 6 months old, male) and porcine (three-crossbred pig <LWD>, one month old, male) stomachs were provided by the Department of Veterinary Anatomy at the University of Miyazaki (Miyazaki, Japan). Equine (thoroughbred, 7 years old, male) stomach samples were obtained from the Equine Research Institute of the Japan Racing Association (Tochigi, Japan). All procedures were performed in accordance with the guidelines of the Japanese Physiological Society for animal care, and the experiments were approved by the University of Miyazaki Animal Experiment Committee (2015–006). Written informed consent was obtained from the owners for the participation of their animals in this study.

Frozen tissues (20 g, 25 g, and 100 g of bovine, porcine, and equine stomachs, respectively) were pulverized and boiled for 10 min in 10 volumes of water to inactivate intrinsic proteases, as described previously (32). The samples were then chilled on ice and adjusted to 1 M acetic acid (AcOH) by adding glacial AcOH. Boiled stomach tissue was homogenized using a Polytron mixer (Kinematica Inc., Lucerne, Switzerland). The crude acid extracts were then centrifuged for 30 min at 10,000 xg. The supernatant was diluted with an equal volume of distilled water and concentrated to about 1/3 amount using an evaporator. The residual concentrate was subjected to acetone precipitation at a 66% acetone concentration. After removing the precipitate, the acetone supernatant was evaporated. It was loaded onto a 10 g Sep-Pak C18 cartridge (Waters), which was pre-equilibrated with 0.1% trifluoroacetic acid (TFA). The cartridge was washed with a 10% acetonitrile (ACN)/0.1% TFA solution and eluted with 100 mL of 60% ACN/0.1% TFA. The eluate was evaporated and lyophilized. The residual materials were redissolved in 1 M AcOH and then adsorbed on an SP-Sephadex C-25 (H + form) chromatography column (Amersham Pharmacia Biotech, Buckinghamshire, UK) pre-equilibrated with 1 M AcOH. Successive elutions with 1 M AcOH, 2 M pyridine, and 2 M pyridine-AcOH (pH 5.0) yielded three fractions: SP-I, SP-II, and SP-III. The lyophilized SP-III fraction, which contained strong basic peptides and ghrelin activity, was separated via CM-ion-exchange HPLC on a TSK CM-2SW column (4.6 x 250 mm; Tosoh) at pH 6.5. The active fractions were further purified using the same column at pH 4.8. The active fractions were diluted in equal volumes of 0.1% TFA and subjected to Sep-Pak Light C18 cartridge (Waters) purification to remove excess salt. The sample was eluted with 60% ACN/0.1% TFA, evaporated ACN, and then separated via reverse-phase (RP)-HPLC using a Symmetry 300 TM C18 column (3.9 x150 mm, Waters) at a flow rate of 1 mL/min on a linear gradient from 10% to 60% ACN/0.1% TFA for 80 min. The eluate was collected in 0.5-mL fractions. An aliquot of each fraction was assayed for ghrelin activity using FlexStation 3. The active fractions were further purified via RP-HPLC using a diphenyl column, 219TP5215 (2.1 x150 mm; Vydac, Hesperia, CA) for 80 min on a linear gradient from 10% to 60% ACN/0.1% TFA at a flow rate of 0.2 mL/min. Each absorption peak was collected, and an aliquot of each fraction was assayed for activity using FlexStation 3. Some peaks were further purified on a Chemcosorb 3-ODS-H column (2.1 x 75 mm, Chemco Scientific, Osaka, Japan) for 80 min. Approximately 20 pmol of the final purified peptide from the main peak was analyzed using a protein sequencer (PPSQ-33A; Shimazu, Kyoto, Japan). One picomole was used for molecular weight determination using MALDI-TOF mass spectrometry (Autoflex III, Bruker).

### 2.3 Peptides

Ghrelin (Human) was purchased from Peptide Institute, Inc. (Osaka, Japan).

## 3 Results

### 3.1 Pharmacological characterization

**Figure 1:**
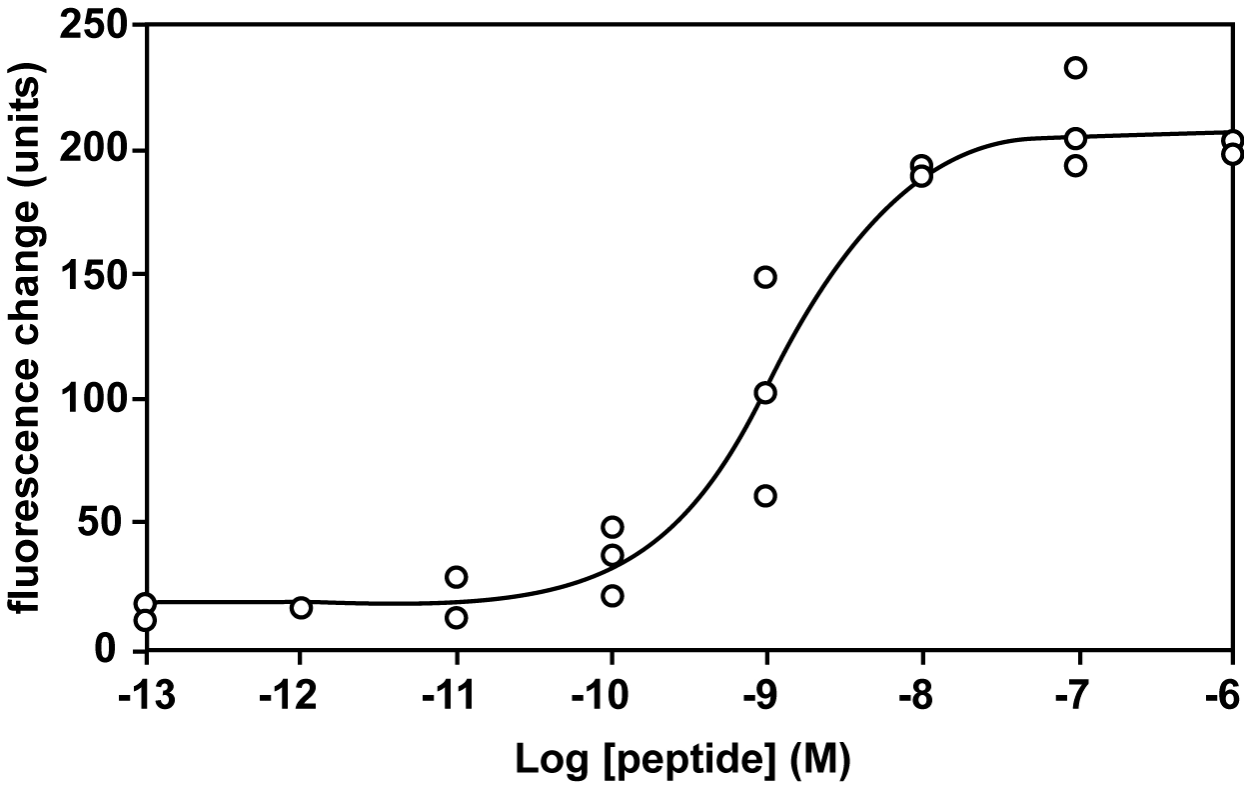
Pharmacological characterization of synthetic human ghrelin using mouse GHS-R1a stably expressed in CHO cells. Dose-response relationships of changes in [Ca^2+^]_i_ for human ghrelin in CHO-mouse GHS-R1a-line 24–5 cells.

The interaction between ghrelin and CHO-mouse GHS-R1a-line 24–5 was examined using synthetic peptides. Human ghrelin induced dose-dependent, robust increases in [Ca^2+^]_i_ in CHO-mouse GHS-R1a-line 24–5, with half-maximal response concentrations (EC_50_) of 1.15 × 10–^9^ M (**Figure 1**). Ghrelin did not induce a response in CHO cells transfected with vector alone (data not shown).

### 3.2 Purification of bovine ghrelin

**Figure 2:**
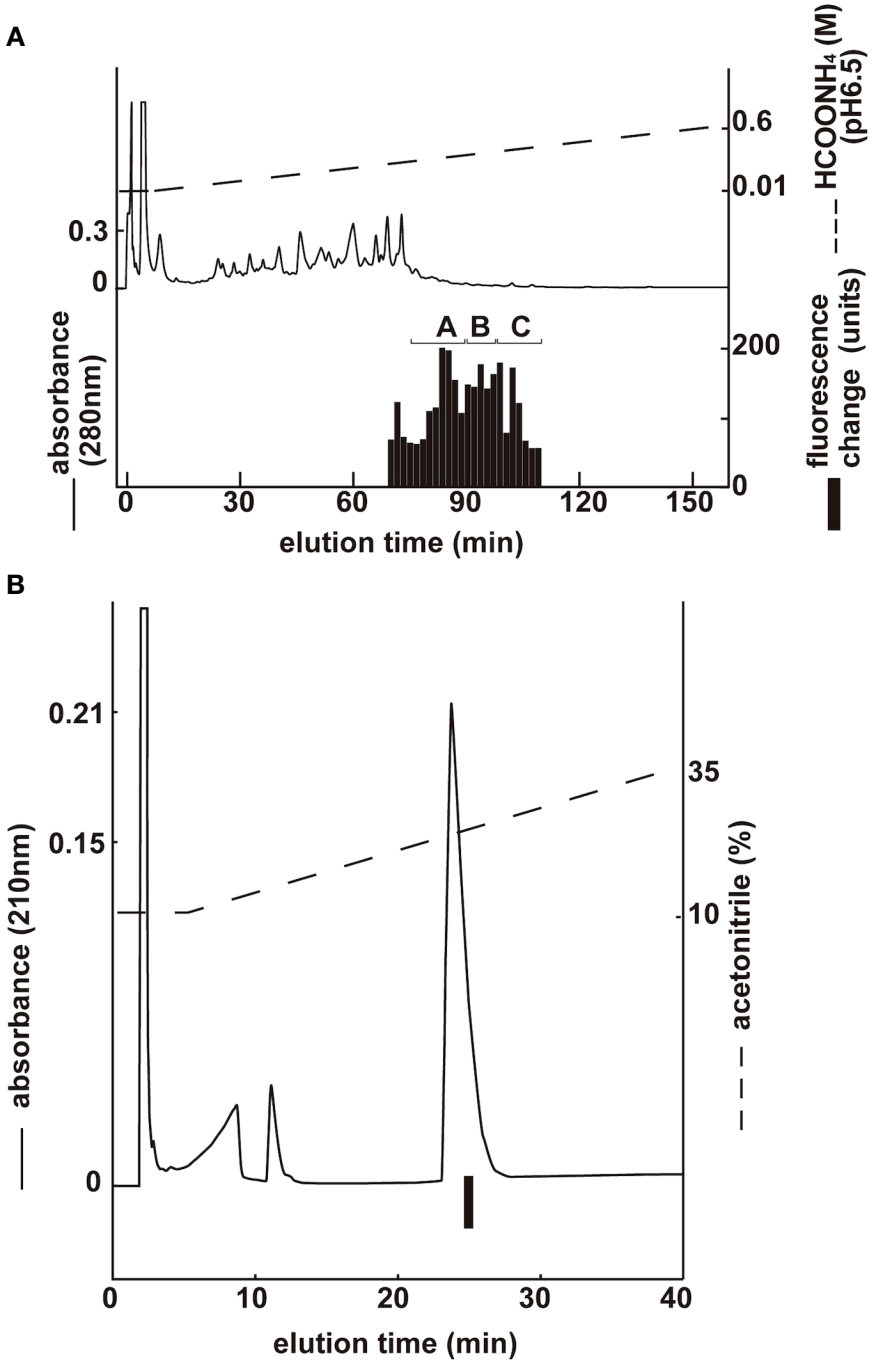
Purification of bovine ghrelin from stomach extract. Black bars indicate the fluorescence changes in [Ca^2+^]_i_ in CHO-mouse GHS-R1a-line 24–5 cells. **(A)** CM ion-exchange HPLC (pH 6.5) of the SP-III fraction of stomach extract. Each active fraction **(A, B)** was subjected to RP-HPLC. **(B)** Final purification of active fraction B from CM-HPLC via RP-HPLC (**Table 1**, peak 2).

Three groups with ghrelin activity were identified via CM ion-exchange HPLC (pH 6.5) of the SP-III fraction (**Figure 2A**). Each active group was purified via CM ion-exchange HPLC (pH 4.8) and three separate rounds of RP-HPLC. The final purification of the group B fractions (**Figure 2A**), which gave the highest ghrelin yield, is shown in **Figure 2B**. Four active peptides were isolated from the three groups using CM-HPLC (**Table 1**). The amino acid sequence of the group B peptide showing the highest activity was determined to be GSXFLSPEHQKLQRKEAKKPSGRLKPR (X was unidentified by the protein sequencer because of an acyl modification). We predicted that X was serine based on the NCBI accession no. NM_174067, and the isolated peptide was identified as a ghrelin peptide based on its sequence homology with other ghrelins. The isolated peptides were analyzed using MALDI-TOF MS to determine the molecular weight of the bovine ghrelins. **Table 1** shows the measured molecular masses, isolation yields, and deduced molecular forms of the isolated peptides. In addition to peptide and genetic information, molecular masses helped in the prediction of two peptide sequences: bovine ghrelin 1–27 (GSSFLSPEHQKLQRKEAKKPSGRLKPR) and bovine ghrelin 1–26 (GSSFLSPEHQKLQRKEAKKPSGRLKP), which lack the last arginine from bovine ghrelin 1–27. The major form of bovine ghrelin was isolated from group B, peak 2, which yielded approximately 367 pmol of peptide. The expected sequence of this peptide was ghrelin 1–27 with n-octanoic acid (C8:0). For some peptides, we could not determine fatty acid modifications because of the inconsistency between the expected and measured masses.

**Table 1:** Expected molecular forms of isolated ghrelins from bovine stomach.

Groups	Peaks	Mass[M+H]	Expected Molecular form	Yields(pmol)
A	1	3059.16	ghrelin-(1–26) (C8:0)	75
B	2	3215.64	ghrelin-(1–27) (C8:0)	367
C	3 4	3230.34 3243.78	ghrelin-(1–27) (C9:0) ghrelin-(1–27) (C10:0)	140 65

### 3.3 Purification of porcine ghrelin

**Figure 3:**
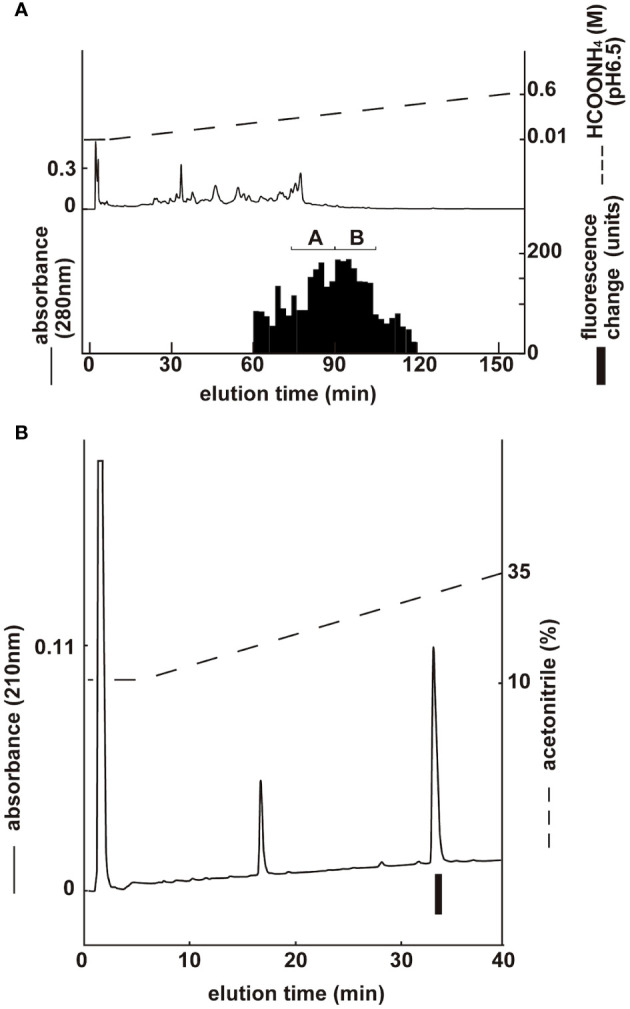
Purification of porcine ghrelin from stomach extract. Black bars indicate the fluorescence changes in [Ca^2+^]_i_ in CHO-mouseGHS-R1a-line 24–5 cells. **(A)** CM ion-exchange HPLC (pH 6.5) of the SP-III fraction of stomach extract. Each active fraction **(A, B)** was subjected to RP-HPLC. **(B)** Final purification of active fraction B from CM-HPLC via RP-HPLC (**Table 2**, peak 2).

Two groups with ghrelin activity were identified via CM ion-exchange HPLC (pH 6.5) of the SP-III fraction (**Figure 3A**). Each active group was purified via CM ion-exchange HPLC (pH 4.8) and three separate rounds of RP-HPLC. The final purification of the group B fractions (**Figure 3A**), which gave the highest ghrelin yield, is shown in **Figure 3B**. Four active peptides were isolated from the two groups using CM-HPLC (**Table 2**). The amino acid sequence of the group B peptide showing the highest activity was determined to be GSXFLSPEHQKVQQRKESKKPAAKLKPR. We predicted that X was serine based on the NCBI accession no. NM_213807.2 The isolated peptide was found to be a ghrelin peptide based on its sequence homology with other ghrelins. To determine the molecular weight of porcine ghrelins, isolated peptides were analyzed using MALDI-TOF mass spectrometry. **Table 2** shows the measured molecular masses, isolation yields, and deduced molecular forms of the isolated peptides. In addition to peptide and genetic information, the molecular masses helped predict two peptide sequences: porcine ghrelin 1–28 (GSSFLSPEHQKVQQRKESKKPAAKLKPR) and porcine ghrelin 1–27 (GSSFLSPEHQKVQQRKESKKPAAKLKP), which lack the last arginine from porcine ghrelin 1–28. The major form of porcine ghrelin was isolated from Group B, peak 2, and these fractions yielded approximately 713 pmol of the peptide. The expected sequence of this peptide was porcine ghrelin 1–28 with n-octanoic acid (C8:0). For some peptides, we could not determine fatty acid modifications because of the inconsistency between the expected and measured masses.

**Table 2:** Expected molecular forms of isolated ghrelins from porcine stomach.

Groups	Peaks	Mass[M+H]	Expected Molecular form	Yields(pmol)
A	1	3159.16	ghrelin-(1–27) (C8:0)	11
B	2 3 4	3315.11 3329.21 3341.55	ghrelin-(1–28) (C8:0) ghrelin-(1–28) (C9:0) ghrelin-(1–28) (C10:0)	713 49 47

### 3.4 Purification of equine ghrelin

**Figure 4:**
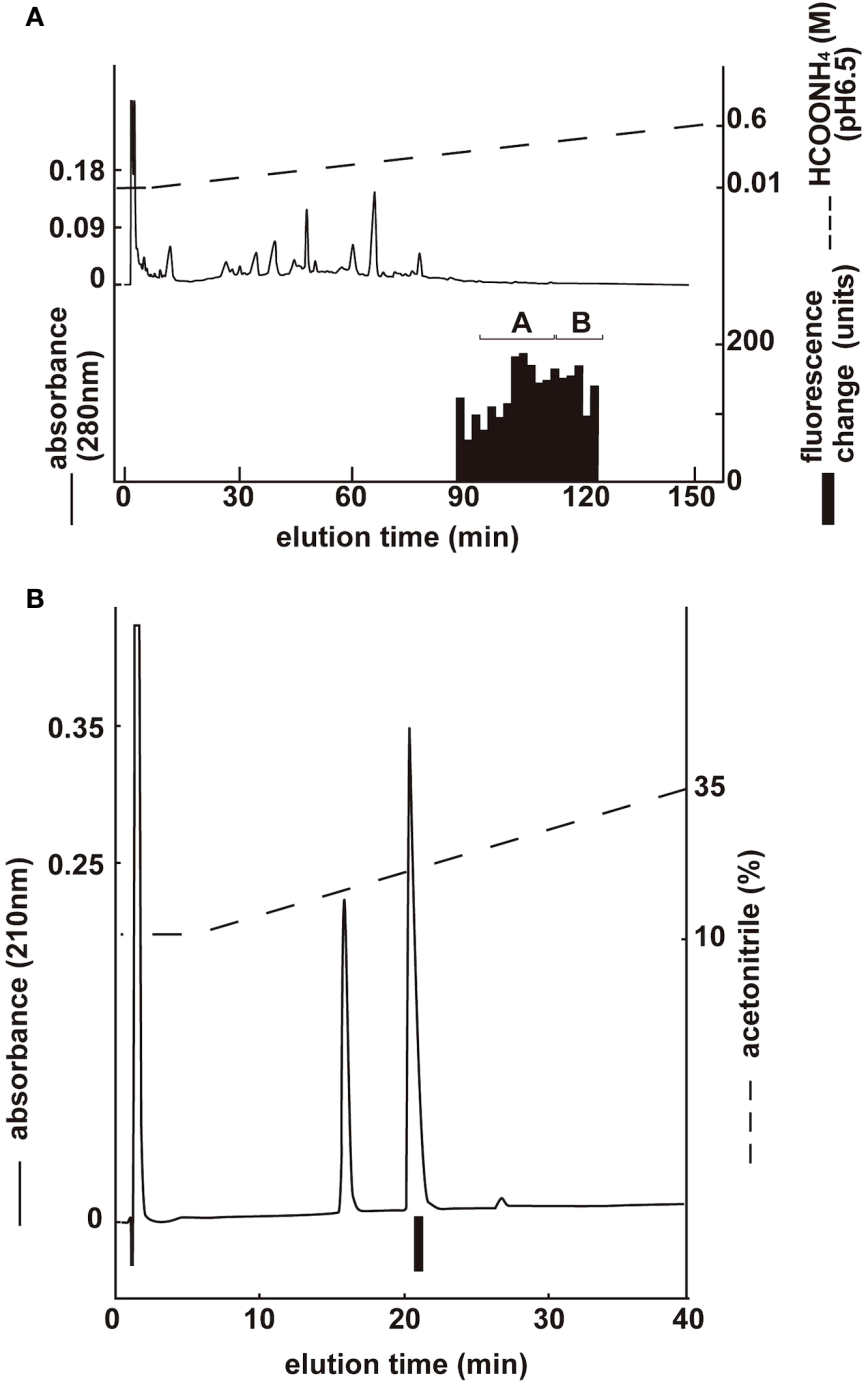
Purification of equine ghrelin from stomach extract. Black bars indicate the fluorescence changes in [Ca^2+^]_i_ in CHO-mouse GHS-R1a-line 24–5 cells. **(A)** CM ion-exchange HPLC (pH 6.5) of the SP-III fraction of stomach extract. Each active fraction **(A, B)** was subjected to RP-HPLC. **(B)** Final purification of active fraction B from CM-HPLC via RP-HPLC (**Table 3**, peak 1).

Two groups with ghrelin activity were identified via CM ion-exchange HPLC (pH 6.5) of the SP-III fraction (**Figure 4A**). Each active group was purified via CM ion-exchange HPLC (pH 4.8) and three separate rounds of RP-HPLC. The final purification of the group A fractions (**Figure 4A**), which gave the highest ghrelin yield, is shown in **Figure 4B**. Six active peptides were isolated from the two groups using CM-HPLC (**Table 3**). The amino acid sequence of the group A peptide showing the highest activity was determined to be GSXFLSPEHHKVQHRKESKKPPAKLKPR. We predicted that X was serine based on the NCBI accession no. XM_023619973: The isolated peptide was a ghrelin peptide based on its sequence homology with other ghrelins. Isolated peptides were analyzed using MALDI-TOF MS to determine the molecular weight of equine ghrelins. **Table 3** shows the measured molecular masses, isolation yields, and deduced molecular forms of the isolated peptides. In addition to peptide and genetic information, the molecular masses helped predict two peptide sequences: equine ghrelin 1–28 (GSSFLSPEHHKVQHRKESKKPPAKLKPR) and ghrelin 1–27 (GSSFLSPEHHKVQHRKESKKPPAKLKPR), which lack the last arginine from equine ghrelin 1–28. The major form of equine ghrelin was isolated from group A, peak 1, and these fractions yielded an approximately 523 pmol of the peptide. The expected sequence of this peptide is equine ghrelin 1–28 with n-butanoic acid (C4:0). For some peptides, we could not determine fatty acid modifications because of the inconsistency between the expected and measured masses.

**Table 3:** Expected molecular forms of isolated ghrelins from equine stomach.

Groups	Peaks	Mass[M+H]	Expected Molecular form	Yields(pmol)
A	1 2	3303.56 3203.81	ghrelin-(1–28) (C4:0) ghrelin-(1–27) (C8:0)	523 16
B	3 4 5 6	3359.73 3373.44 3385.72 3387.80	ghrelin-(1–28) (C8:0) ghrelin-(1–28) (C9:0) ghrelin-(1–28) (C10:1) ghrelin-(1–28) (C10:0)	139 75 63 14

## 4 Discussion

In the present study, we determined the amino acid sequences of bovine, porcine, and equine ghrelins and the fatty acid modifications in these ghrelin peptides. The major active form of bovine ghrelin was a 27-amino acid peptide with an n-octanoyl modification at Ser^3^ because of its yield in the stomach. The major active form of porcine and equine ghrelin was a 28-amino acid peptide. However, porcine ghrelin was modified with n-octanol, whereas equine ghrelin was modified with n-butanol at Ser^3^.

**Figure 5:**
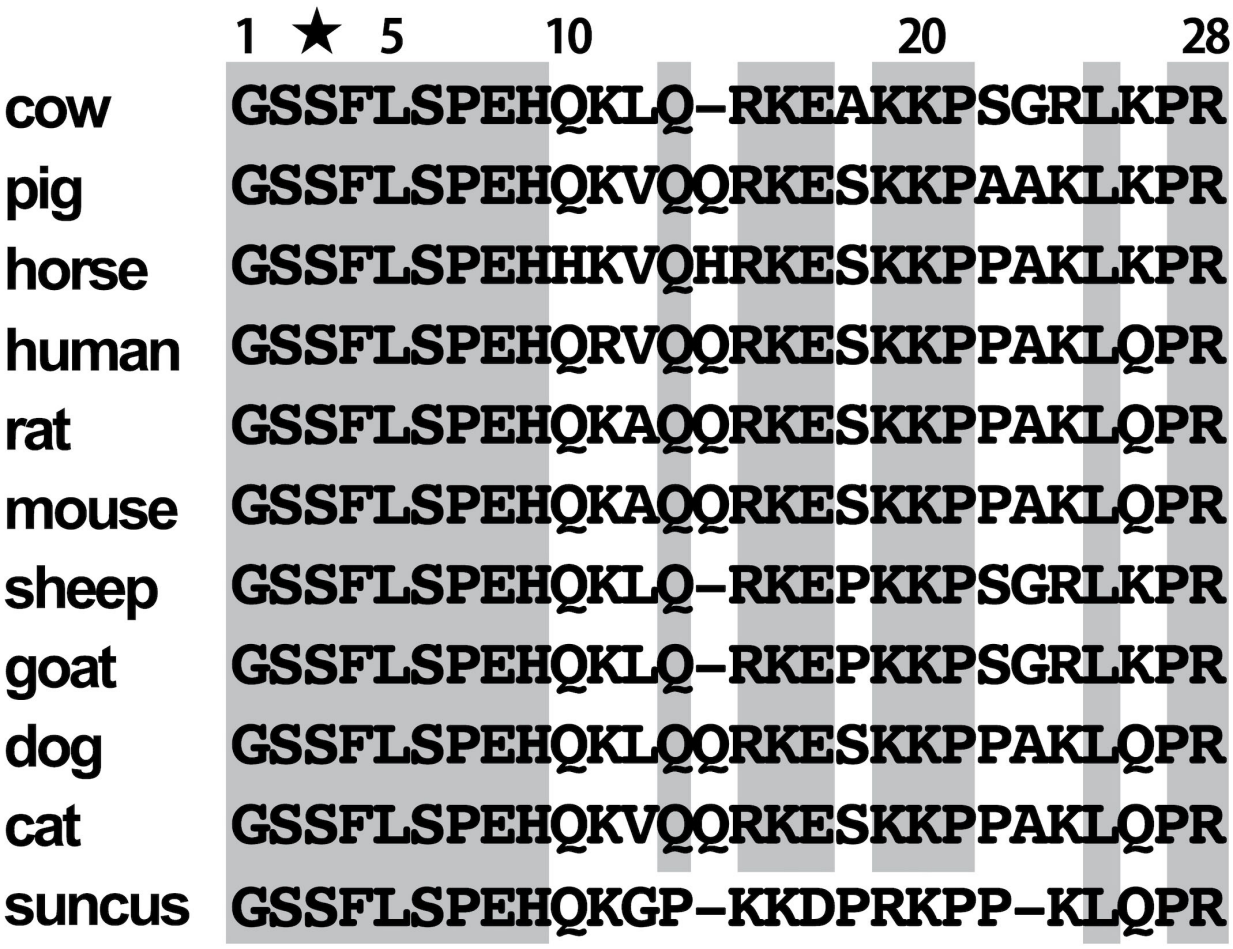
Amino acid sequence comparison of mammalian ghrelin. An asterisk indicates Ser^3^ modified by fatty acid.

Similar to porcine and equine ghrelin, mammalian ghrelin is composed of 28 amino acids in general (1). Otherwise, bovine ghrelin identified in this study lacks Gln^14^ that is present in rat and human ghrelin (30, 31). Gln^14^ deletion has been observed in other mammals, such as sheep and goats (33). Des-Gln^14^-ghrelin has been known to be a second ligand for GHS-R1a in rat and human, and it is generated by alternative splicing of the ghrelin gene (30). However, in suncus, genomic analysis of the intron and exon boundaries of mature ghrelin revealed that the last nucleotide triplet in the second intron did not code for Gln, indicating that alternative splicing does not occur in the same manner as in rats (34). Ruminants may not have alternative splicing machinery for des-Gln^14^-ghrelin production. As there is no Gln^14^ in ruminants, the mechanism is suspected to be the same as that in suncus (**Figure 5**).

The major forms of bovine and porcine ghrelin were modified by n-octanoic acid (C8:0), constituting approximately 51% and 69% of all purified ghrelin, respectively. Similar to the previous results of purifying caprine ghrelin, n-nonanoylated (C9:0) and n-decanoylated (C10:0) forms of bovine ghrelin were identified, accounting for 20% and 9% of the total purified ghrelin, respectively. The most notable difference between equine ghrelin and ghrelins from other species was the presence of n-butanoic acid (C4:0)-modified form (approximately 40% purified ghrelin). This peptide was confirmed to be ghrelin based on fluorometric assay results, which indicated increased [Ca^2+^]_i_ in rat GHS-R1a-expressing cells (CHO-mouse GHS-R1a-line 24–5 cells). These purified molecular forms of ghrelin were predicted, as shown in **Tables 1**–**3**. Interestingly, the n-butanoylated (C4:0) form of ghrelin, the major form found in horses, has not yet been reported in other species. Recently, several GHS-R1a structures have been identified, including the active ghrelin-binding form (35–37). The ligand-binding pocket of GHS-R1a had a unique architecture, a pocket bifurcated into two cavities not found in closely related GPCRs, and this structural feature was known to be utilized to recognize the octanoic acid modification of ghrelin. There were no differences in the amino acid sequences of these cavities, including the most important amino acids E124 and R283, between equine and human GHS-R1a (NM_001494000.3 and NM_198407.2). The activity of the n-butanoylated (C4:0) form of equine ghrelin in rat GHS-R1a-expressing cells was significantly lower than of equine ghrelin 1–28 (C8:0) (data not shown). Therefore, even in horses, the physiological effects of ghrelin are thought to be more pronounced for ghrelin modified by n-octanoic acid (C8:0) than by n-butanoic acid (C4:0). It remains unclear why ghrelin modified by n-butanoic acid (C4:0) is the main form in horses; however, in thoroughbreds, the feeding-enhancing effect of ghrelin may be suppressed. In other words, because running is more important than growth, the less active ghrelin modified by n-butanoic acid (C4:0) may be dominant.

The acyl modification of ghrelin is catalyzed by GOAT (25). It is likely that ghrelin is modified by GOAT in cows, pigs, and horses; however, the underlying mechanism by which ghrelin is modified with various fatty acids remains unknown. Feeding conditions or composition may influence the type and extent of acyl modification of ghrelin (38). Currently, ghrelin is measured in many animals to elucidate its physiological functions or apply it to animal models. However, most measurements are performed using the commercial active ghrelin ELISA or RIA kit. These “active ghrelin” ELISA or RIA kit measures human ghrelin- (1–28) (C8:0) (39, 40). Based on a comparison of the structures of bovine, porcine, equine, and human ghrelins, the kit appears to be suitable for the measurement of ghrelins modified by n-octanoic acid (C8:0), although it remains unknown whether other ghrelin-derived molecules can be recognized using this procedure. Therefore, it remains unclear whether the concentrations measured using commercial ELISA and RIA kits were accurate. One solution is to use ghrelin C-terminal antibodies from each species and RP-HPLC. This study revealed the elution position of each modified form of ghrelin in RP-HPLC. Therefore, by developing the plasma using RP-HPLC and measuring each fraction with a ghrelin C-terminal antibody for each species, the exact amount of each ghrelin-modified form can be determined. Future studies should investigate the effects of both major and minor forms of ghrelin on its physiological roles. In order to elucidate more precise physiological effects of ghrelin in each animal species, it is necessary to conduct experiments on the administration of various acyl modifications of ghrelin and to analyze the detailed tissue distribution of GHS-R1a.

## Data availability statement

The datasets presented in this study can be found in online repositories. The names of the repository/repositories and accession number(s) can be found in the article/supplementary material.

## Ethics statement

The animal studies were approved by University of Miyazaki Animal Experiment Committee (approval no.2015-006). The studies were conducted in accordance with the local legislation and institutional requirements. Written informed consent was obtained from the owners for the participation of their animals in this study.

## Author contributions

TI: Investigation, Writing – original draft, Writing – review & editing, Funding acquisition, Visualization. HT: Data curation, Investigation, Writing – original draft. EI: Data curation, Investigation, Writing – original draft. AkK: Investigation, Writing – original draft. AO: Investigation, Writing – original draft. KS: Investigation, Writing – original draft. JK: Supervision, Writing – original draft. AtK: Resources, Writing – original draft. HO: Resources, Writing – original draft. SN: Data curation, Writing – original draft. KK: Data curation, Writing – original draft. TY: Supervision, Writing – original draft. MS-H: Data curation, Writing – original draft. MY: Resources, Writing – original draft. MM: Supervision, Writing – original draft. YS: Data curation, Writing – original draft. TS: Funding acquisition, Writing – original draft. MK: Funding acquisition, Writing – review & editing, Writing – original draft.
